# ARAM: an automated image analysis software to determine rosetting parameters and parasitaemia in Plasmodium samples

**DOI:** 10.1186/s12936-016-1243-4

**Published:** 2016-04-18

**Authors:** Patrick Wolfgang Kudella, Kirsten Moll, Mats Wahlgren, Achim Wixforth, Christoph Westerhausen

**Affiliations:** Experimental Physics I, University of Augsburg, Universitätsstraße 1, Augsburg, Germany; Department of Microbiology, Tumor and Cell Biology, Karolinska Institutet, Box 280, 171 77 Stockholm, Sweden; Nanosystems Initiative Munich, Schellingstraße 4, Munich, Germany

**Keywords:** Malaria, *Plasmodium falciparum*, Parasitaemia, Rosetting, Automatic analysis, Software, Image analysis, Cell detection

## Abstract

**Background:**

Rosetting is associated with severe malaria and a primary cause of death in *Plasmodium falciparum* infections. Detailed understanding of this adhesive phenomenon may enable the development of new therapies interfering with rosette formation. For this, it is crucial to determine parameters such as rosetting and parasitaemia of laboratory strains or patient isolates, a bottleneck in malaria research due to the time consuming and error prone manual analysis of specimens. Here, the automated, free, stand-alone analysis software automated rosetting analyzer for micrographs (ARAM) to determine rosetting rate, rosette size distribution as well as parasitaemia with a convenient graphical user interface is presented.

**Methods:**

Automated rosetting analyzer for micrographs is an executable with two operation modes for automated identification of objects on images. The default mode detects red blood cells and fluorescently labelled parasitized red blood cells by combining an intensity-gradient with a threshold filter. The second mode determines object location and size distribution from a single contrast method. The obtained results are compared with standardized manual analysis. Automated rosetting analyzer for micrographs calculates statistical confidence probabilities for rosetting rate and parasitaemia.

**Results:**

Automated rosetting analyzer for micrographs analyses 25 cell objects per second reliably delivering identical results compared to manual analysis. For the first time rosette size distribution is determined in a precise and quantitative manner employing ARAM in combination with established inhibition tests. Additionally ARAM measures the essential observables parasitaemia, rosetting rate and size as well as location of all detected objects and provides confidence intervals for the determined observables. No other existing software solution offers this range of function. The second, non-malaria specific, analysis mode of ARAM offers the functionality to detect arbitrary objects.

**Conclusions:**

Automated rosetting analyzer for micrographs has the capability to push malaria research to a more quantitative and statistically significant level with increased reliability due to operator independence. As an installation file for Windows © 7, 8.1 and 10 is available for free, ARAM offers a novel open and easy-to-use platform for the malaria community to elucidate rosetting.

**Electronic supplementary material:**

The online version of this article (doi:10.1186/s12936-016-1243-4) contains supplementary material, which is available to authorized users.

## Background

The most deadly animals worldwide are mosquitoes as they are the main transmitter of malaria parasites. In the year 2014 about 220 million malaria cases occurred globally, while about 430,000 people died due to a malaria infection [1]. There are five species of the malaria parasite *Plasmodium* that can infect humans: *Plasmodium falciparum*, *P. vivax*, *P. ovale*, *P. malariae* and *P. knowlesi*, whereof *P. falciparum* leads to the highest severity of disease and number of deaths. Its survival in the human host depends on the ability to adhere to endothelial cells which allows sequestration in the microvasculature and thus to avoid splenic clearance [2, 3]. The formation of rosettes, a mechanism where parasitized red blood cells (pRBC) bind to two or more unparasitized red blood cells (RBC), leads to formation of cell aggregates. These aggregates, in turn, are discussed to facilitate the sequestration of the pRBC and consequent obstruction of capillaries and are suggested to be associated with severe malaria [3–6]. Even though there is a number of studies on the molecular mechanisms of rosetting using in vitro cultures identifying the crucial receptors and developing anti rosetting drugs [7–16], the mechanism and function of rosetting is not fully understood to date. Important parameters necessary to analyse and research with focus on the rosetting phenomenon are the parasitaemia *P* (the ratio of pRBC to total RBC count)

1 $$P = N_{\text{pRBC}} /N_{\text{RBC}}$$

the rosetting rate R (the fraction of pRBC binding two or more other RBC)

2 $$R = N_{\text{rosettes}} /N_{\text{pRBC}}$$

and the size distribution of the rosettes.

To date the standard procedure to determine rosette rate and size is manual counting of a stained culture in the microscope. This is time consuming and susceptible for operator dependent systematic errors. Especially when working with anti-rosetting drugs requiring large number of samples the manually performed analysis is a bottleneck. For the significance of the findings in all studies the objectivity of the operator and a sufficiently high number of analysed RBC is crucial. To reduce analysis time and improve the uniformity as well as accuracy of the results automation of the whole process is highly desirable.

A very efficient way to determine the parasitaemia of a specimen is to use fluorescence activated cell sorting (FACS). The determination of parasitemia only with the help of FACS is automated, fast and possibly to prefer if access to a FACS is granted, but the set-up is very costly and also expensive to maintain.

Another way is the fully automated analysis of microscope images. There are several publications describing software and automated algorithms to determine at least one of the before named measures at a time. One of the first approaches of automatic image analysis of stained blood smears is the MATLAB ^®^ -based script *MalariaCount*, developed by Sio et al. [17, 18]. The analysis depends on command line input as the tool has no graphical user interface (GUI). Furthermore, the script needs an installation of MATLAB ^®^. *MalariaCount* only gives parasitaemia as result. The source code is available on the journal website. With a three-step algorithm Dìaz et al. [19] determine parasitaemia and the parasites infection stage. This algorithm needs image preprocessing, color images for segmentation and a set of classifiers to identify the erythrocytes infection stage. Unfortunately, it is not mentioned how this software is implemented or where to get its source code. Frean uses the free software *ImageJ* [20] in combination with a script to determine parasitaemia in thick Giemsa-stained blood smear microscope images [21]. Therefore, he combined the particle counting algorithm of *ImageJ* and tunes its parameters in a semi-automatic way. His script is available in the publication. Tek et al. [22] gives an overview about literature concerning automated blood film analysis for malaria and similar infectious diseases.

Despite these publications a great number of malaria research laboratories still estimates parasitaemia and rosetting rate by manual counting of pRBC and RBC. This is due to a lack of a capable, convenient, free software that is easy to operate.

In this paper a free, easy-to-use, stand-alone software that allows to detect parasitaemia, rosetting rate and rosette size distribution via a self-explaining, graphical user interface is presented. The automated rosetting analyzer for micrographs (ARAM) analysis itself is performed automatically, significantly faster than by hand and without additional operator input or preprocessing of the images. Additionally, in contrast to FACS, ARAM offers the possibility to check the detection results on each analysed frame.

## Methods

### Cell culture

*Plasmodium falciparum* laboratory strains are cultivated according to standard protocols [23] under shaking conditions during cultivation. By enrichment in a Ficoll-gradient solution or through enrichment with monoclonal antibodies (mAbs) [23] the rosetting phenotype is maintained.

### Image acquisition and analysis

#### Sample preparation

For the manual analysis the parasite culture is stained with 10 µg/ml of acridine orange for 5 min at room temperature (RT). For analysis with ARAM the same preparation process is used but the parasite culture gets diluted to a haematocrit of 0.2 % to prevent cell overlapping. In addition, the slides are left at RT for a total of 10 min to allow the cells to settle down prior to microscopic investigation.

#### Inhibition assays

In rosette disruption assays 45 µl of cultures are mixed with 5 µl of heparin (10× final concentration) and are then incubated at room temperature for 60 min. For the mixing wide pipettes tips are used to not mechanically interfere with rosettes.

#### Image acquisition routine

In the manual analysis 500 RBC are counted for parasitaemia and 100 pRBC for rosetting rate determination like described in [23]. Therefore, a Nikon Optishot-2 microscope with 40× objective is used. Neighboring fields are selected for counting all parameters by moving from the upper left corner of the slide to the lower right and from the upper right corner to the lower left corner to ensure compensation for potential uneven distribution of pRBC and rosettes on the slide. For the ARAM analysis a Nikon Eclipse80i microscope with 40× objective is utilized. The images are captured by a Hamamatsu ORCA ER camera with NIS element software. The same neighbouring fields selection method as described above is used and 50 or 100 images are taken with a FITC filter with simultaneously applied bright light (exposure time 1 s). For further analysis, the images are exported in jpg-file format.

### Software

With the goal of creating a free, stand-alone application MATLAB ^®^ is chosen as the underlying software framework. The collection of scripts that make up ARAM is then compiled and accessible as executable file. This allows ARAM to run without a MATLAB ^®^ installation. The basic concept in detecting objects on the images is to separate them from the background. Therefore, an algorithm transforms the image into a binary map of the original, where pixels that are part of the cell object are white, and background pixels are black. There are different methods to identify objects. In default mode ARAM works with a derivation-based algorithm to detect parasitaemia, rosetting rate and rosette size distribution from micrographs with fluorescent pRBC. A second operation mode is included which can use an additional threshold based algorithm to detect size distribution and location of arbitrary objects, such as nano-particles or vesicles. During development of the software a third background based algorithm for object identification was created but is not implemented in ARAM: as this algorithm utilizes the most apparent procedure it is described in the following paragraph to explain why it does not meet the requirements of an automated detection method.

### Background-based algorithm

To separate cell objects from the background this algorithm creates a background-only version of the analysed image by line wise fitting of intensity values, that is subtracted from the original image. To account for non-conformity of illumination in typical micrographs the algorithm applies two threshold operations near the mean intensity of the image to isolate the background. Areas that do not meet the threshold range are filled with the mean intensity value. The resulting matrix is line wise fitted with a polynomial to create a smooth background-only image that includes non-uniform illumination. The algorithm subtracts this new background-version from the original image leaving a black background and lighter cells (as shown in Additional file 1: Figure S1). For highly non-uniform backgrounds the algorithm is slowed down due to the great number of necessary polynomial fits.

### Threshold-based algorithm

For very uniformly lit backgrounds and high contrast between objects and background a threshold-based algorithm delivers the best object detection results. Therefore, every pixel of the grey value image is compared to a threshold value: pixels with higher intensity are displayed white, pixels with lower intensity are displayed black. The analysis part of the algorithm then searches the resulting binary image for the detected objects. An example for this algorithm is shown in the Additional file 2: Figure S2. ARAM optionally uses this algorithm in the object counting mode but not in the default mode as the background is lighter than the cells but darker than the pRBC, which would need two threshold operations simultaneously.

### Gradient based algorithm

This algorithm detects object contours in an image through the local derivation of neighboring pixel values. Therefore, RGB-colored micrographs are transformed to 8-bit grey value images. As the standard edge detection procedure the algorithm uses the Prewitt filter with the Prewitt operator as the kernel of this filter. This operator determines the gradient in x-direction and y-direction of the image. With the original image **A** the vertical and horizontal operators are

3 $${\mathbf{G}}_{x} = \left[ {\begin{array}{*{20}c} { - 1} & 0 & {{ + }1} \\ { - 1} & 0 & {{ + }1} \\ { - 1} & 0 & {{ + }1} \\ \end{array} } \right]*{\mathbf{A}} ,$$

$${\mathbf{G}}_{y} = \left[ {\begin{array}{*{20}c} { + 1} & { + 1} & { + 1} \\ 0 & 0 & 0 \\ { - 1} & 1 & { - 1} \\ \end{array} } \right]*{\mathbf{A}}$$

The operation * represents the 2D convolution of kernel **k** (the matrix in Eq. 3) and image **A**. Because the matrices are not continuous functions the discrete formulation of a 2D convolution is utilized:

4 $$\begin{aligned}{\mathbf{G}}_{i} \,\left( {x,y} \right) &= {\mathbf{k}}_{i} \,\left( {m,n} \right) \otimes {\mathbf{A}}\,\left( {x,y} \right)\\&=\sum_{m = - 1}^{1} \sum_{n = - 1}^{1} {\mathbf{k}}_{i} \;\left( {m,n} \right){\mathbf{A }}\;(x - m,y - n) \nonumber\end{aligned}$$

For every pixel the algorithm calculates the gradient magnitude from both contributions in Eq. (3) as

5 $${\mathbf{G}} = \sqrt {{\mathbf{G}}_{x}^{2} + {\mathbf{G}}_{y}^{2} }$$

**Fig. 1:**
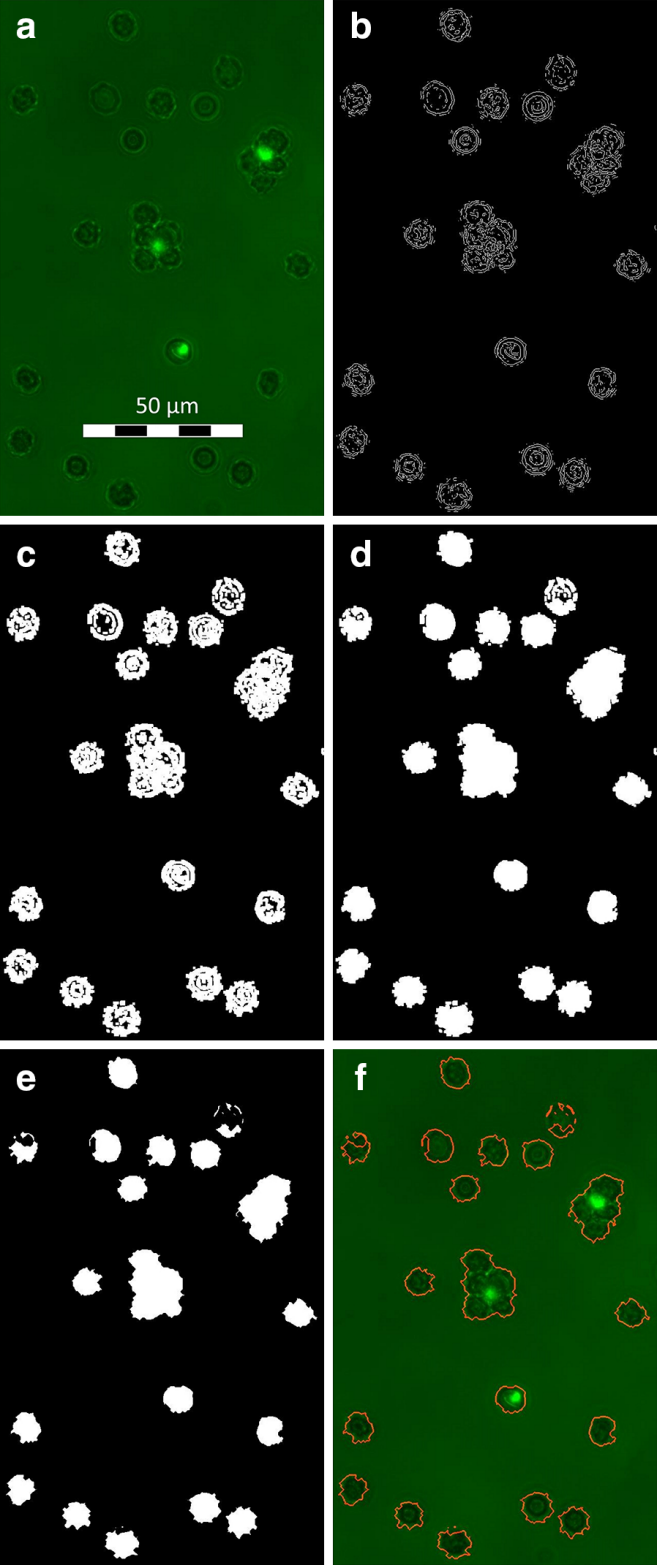
Gradient based algorithm. **a** The original micrograph with two rosettes, one single pRBC and multiple healthy RBC. **b** The Prewitt filter is applied and the threshold of the resulting gradient map displayed. **c** The edges from **b** are dilated like shown in Fig. 2. **d** Capsuled parts of the cells are filled. **e** The *diamond shaped* erosion filter shrinks the outline of the marked areas to a size comparable to the before detected outermost edges. **f** The edges are taken as outlines and plotted onto the original image for better visualization

**Fig. 2:**
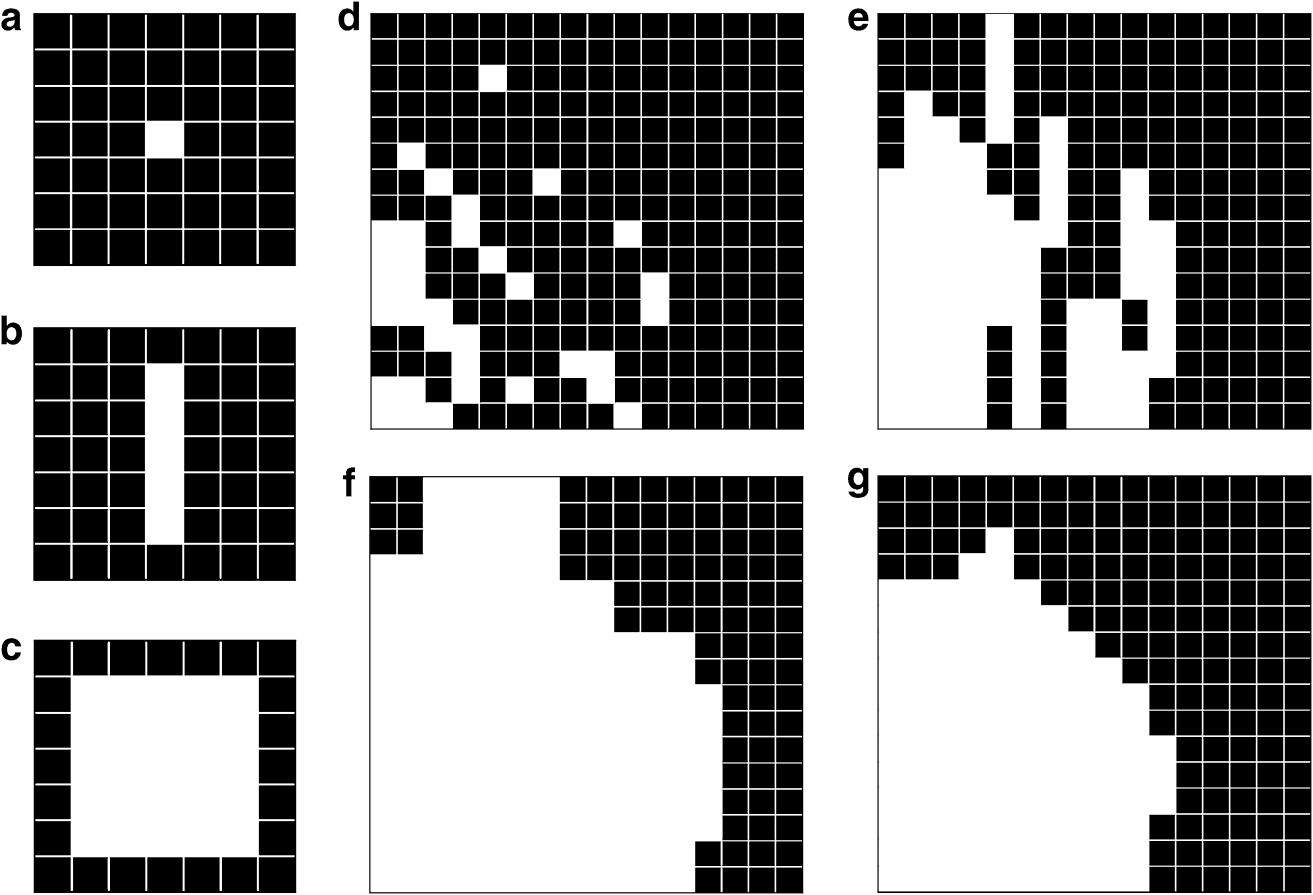
Schematic cell boarder dilation. The single *white pixel* in image **a** gets dilated in two steps: **b** first in vertical direction by a factor of five (**c**) and this operation is applied to the dilated image in horizontal direction also with a factor of five. In **d** a typical segment of the binary detected edges image is shown, in **e** a vertical dilation of all pixels (factor five) and in **f** an horizontal dilation of all pixels (factor five). **g** The eroded *white areas*. The suggest outline from the *top left* image is now clearly visible

The result is a matrix of derivative approximations for every pixel, where a threshold filter creates binary entries from the calculated magnitude values. The background is now black (0) and the found edges white (1). In Fig. 1(b) a typical result is shown. Detected edges are marked as points and stripes with a width of one pixel. A dilation of these white structures in horizontal and vertical direction, as depicted in Fig. 1c, connect the whole cell boundary (adjustable in the configuration file *expansion factor for cell detection*; default value: 5). In Fig. 2, the process is schematically displayed for a single point and for multiple lines. To fill enclosed areas within the cell wall outline the algorithm uses the MATLAB ^®^ function *imfill*. The so detected and marked cell areas are bigger than indicated by the edge detection filter. A correction is applied by an erosion filter with a diamond-shaped structuring element of tunable size (adjustable in the configuration file *factor for adjusting dilation in cell detection*; default value: 2). This filter skims white pixels on the 2D-surface of the areas. The resulting detected objects render the cells in the original image, as shown clearly in Fig. 1f.

The advantages of this algorithm are the ability to handle a wide range of object shapes, sizes and structure without the need for an image dependent pre-processing. For images with pronounced noise a blur- or smooth-preprocessing step helps to avoid erroneous edge-detections. Since ARAM is intended to be fast, applicable to all kinds of images without significant preprocessing and without the operator input the *gradient*-*based algorithm* is used for detecting cell objects on micrographs as default detection algorithm in both above named operation modes.

### Detecting parasitized RBC and rosettes

For the detection of pRBC ARAM uses another routine. As mentioned above, labelling of pRBC with a fluorescent dye is established and the most common method for pRBC detection [23]. The signal of the pRBC is usually much brighter than the background and RBC. To identify the pRBC a simple threshold operation is sufficient and produces a binary image with white pRBC on black background. ARAM detects rosettes based on the size of a single cell (*SCS*). An agglomerate of minimum size *m* (adjustable in the configuration file *rosettes cut*-*off size factor*; default value: 2.3) containing at least on pRBC is defined as a rosette:

6 $${\text{detected area}} \left\{ { \begin{array}{*{20}ll} { {<m} \times SCS = {\text{RBC}}} \\ { {\ge m} \times SCS = {\text{rosette}}} \\ \end{array} } \right.$$

If there is more than one pRBC in a cluster, it is still counted as one rosette. For identifying rosettes ARAM calculates the centroid for all previously found pRBC. It then compares these coordinates with the detected cells in the first analysis step. If the detected area on the original picture around the coordinates fulfils the condition above, it is filled with the background color. ARAM applies an inversion of the image and subtracts that from the original cell binary image to get a binary image delivering the result image of the identified rosettes. This new image sequence is analysed along to the object counting step. The result window of ARAM presents an overview of all determined observables like cell and pRBC count, rosette count and size distribution as well as parasitaemia with further statistical information on the confidence intervals. Furthermore, the export function creates a text file with all data including a table of all detected objects, e.g. for further statistical processing.

To assure the quality of the results ARAM performs an error analysis. Uncertainties in the analysis are small compared to the influence of statistical deviations in e.g. cell size. Since a few microscope images with a small amount of cell objects do not represent the entire population of cells and cell size distribution in the sample a statistical approach is necessary. This way it is possible to quantify the impact of non-uniform cell and rosette distribution throughout the specimen. Additionally, the software does not have algorithm induced errors and detects 100 % of objects and observables in ideal images. A measurement or algorithm error would depend on the analysed image series and is hard to quantify. Therefore, ARAM calculates error margins ε for a given confidence probability.

The found result lays in the calculated error margin of width 2ε:

7 $$\begin{aligned} \varepsilon &= \phi^{ - 1} \left( {1 - \frac{1}{2} \times (1 - \varGamma )} \right) \times \left( {Z \times \left( {1 - Z} \right)} \right)^{1/2} \\ &\quad \times n^{{ -{1/2}}} \times \left( {1 - \frac{{\left( {n - 1} \right)}}{{\left( {N - 1} \right)}}} \right)^{1/2}\end{aligned}$$

with the x-quantile of the standard normal distribution *ϕ*^−1^ (x), the chosen confidence probability *Γ*, the calculated quantity of interest Z (like parasitaemia), sample size n and the population size N [24]. Equation 7 holds without restrictions if the following conditions apply:

8 $$Z \times n \ge 50$$

9 $$\left( {Z \times n \times \left( {1 - Z} \right) \times \left( {1 - \frac{n - 1}{N - 1}} \right)} \right)^{1/2} > 3 .$$

by increasing the sample size n this can be ensured.

## Results and discussion

**Fig. 3:**
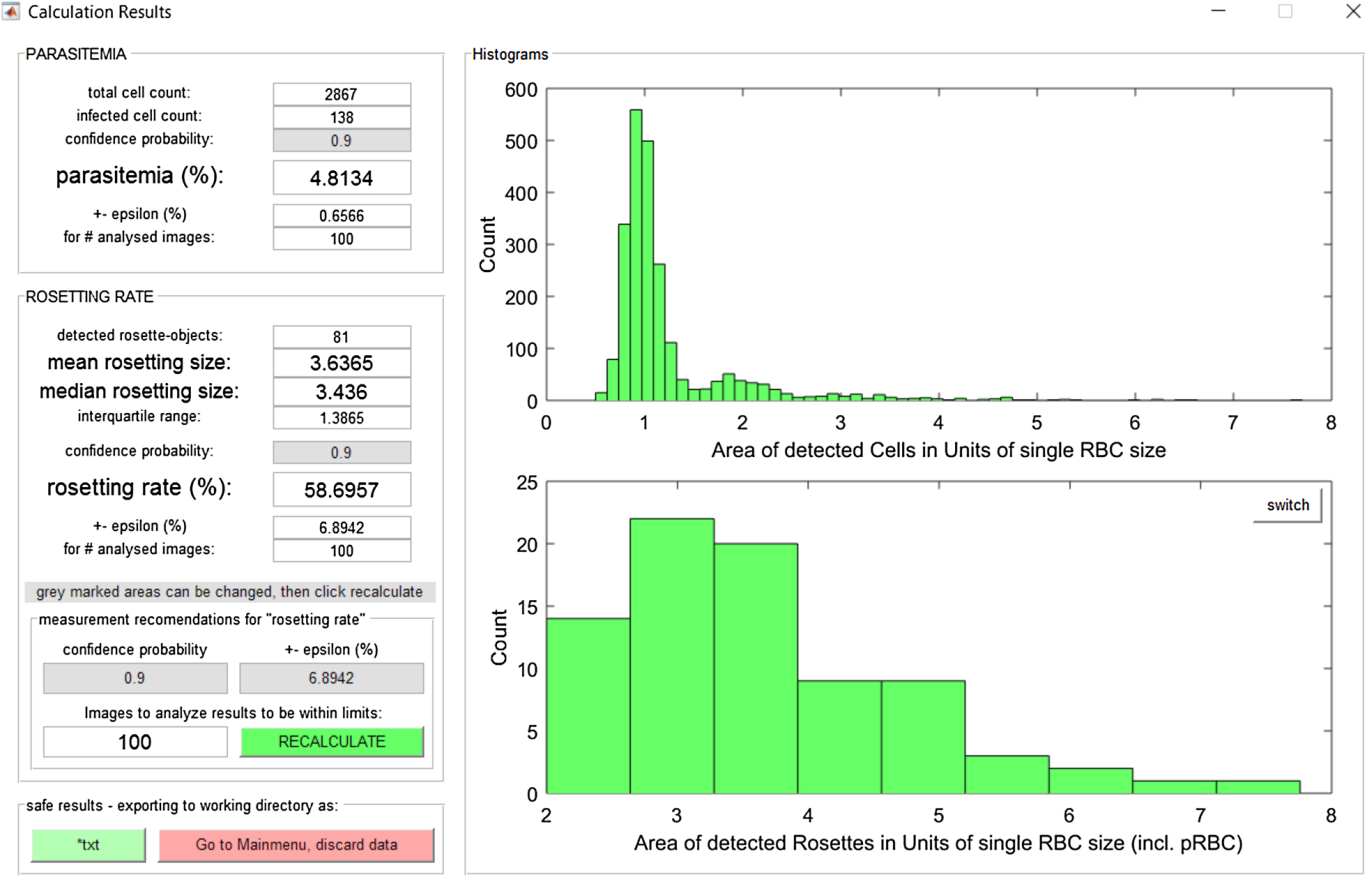
ARAM results window. The ARAM results window gives the most important calculated values. The *green button* on the *bottom left* exports all data to a text file in the working directory. The histogram in the *top right* is a typical object size distribution from a 100 images dataset. Two peaks are apparent, the greater one marks the single cell size area

ARAM presents all determined parameters in a results window shown in Fig. 3, displaying numerical values for parasitaemia, rosetting rate and rosette size as well as bar charts for object size distribution and rosette size distribution in units of a single RBC cell size. All values in the following discussion are taken from ARAM results window or text export.

### Validation of ARAM

**Fig. 4:**
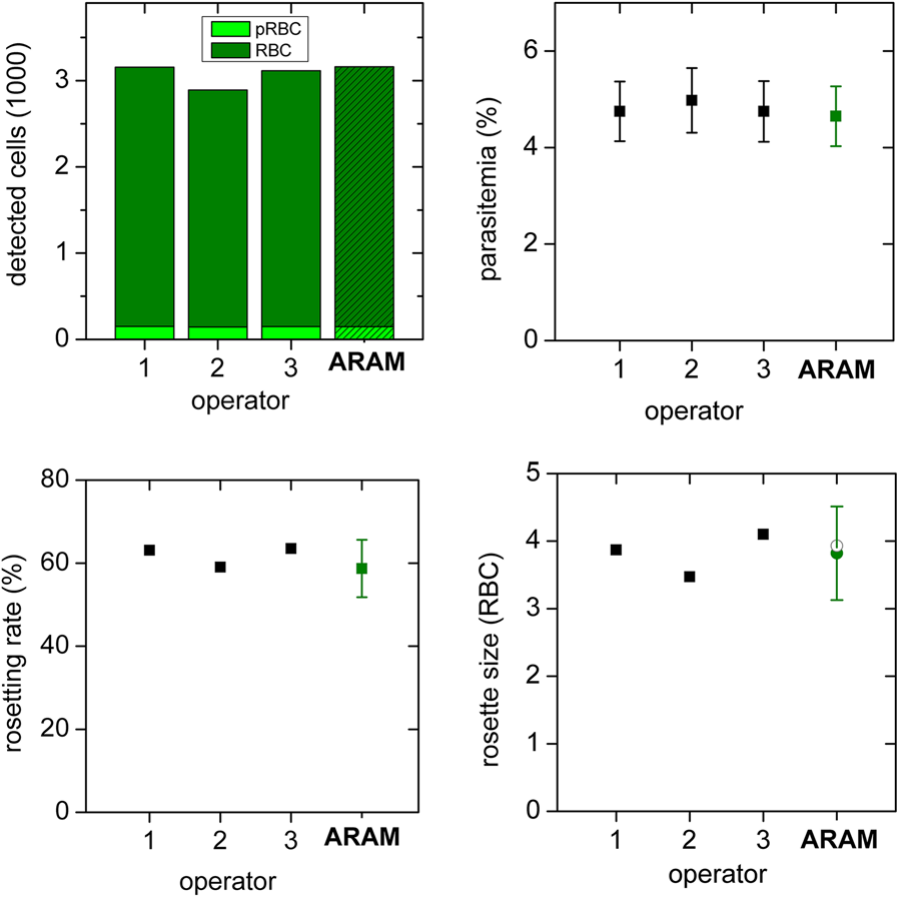
Standard experiment results. *Top left* count of detected cell (RBC) and infected cells (pRBC) in 100 images: the blank graphs are manually counted by three different operators, the *hatched bars* are the counts detected by the software. *Top right* data of *top left* graph convoluted to parasitaemia. Results from ARAM are similar to the manual analysis results. The error bars mark the statistical error (90 % confidence interval). *Bottom left* rosetting rate of manual analysis falls within the statistical error of ARAM’s result. *Bottom right*: Rosette size is given as the average size out of all detected rosettes. The *error bar* from ARAM gives the interquartile range, the *grey circle* the median value

**Fig. 5:**
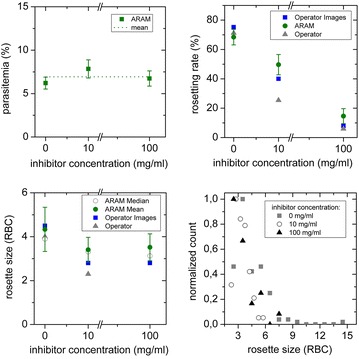
Inhibition experiment results. Samples are treated with three different concentrations of inhibitor, compared are results generated by ARAM, operator on images and operator in microscope. *Top left* parasitaemia: values are similar and their *statistical errors* overlap. *Top right* rosetting rate: manual and automated analysis of micrographs correlate better than different manual analysis of the same sample. *Bottom left* rosette size: the *error bar* on the ARAM results gives the interquartile range, the *grey circle* the median value. *Bottom right* ARAM analysis of rosette size of individual rosettes from samples with different amount of inhibitor delivers a rosette size distribution

To characterize ARAM’s capability to analyse parasitaemia, rosetting rate and rosette sizes in comparison to manual analysis, 100 images are taken from a specimen and analysed by independent operators (in Figs. 4, 5 shown as operator 1–3) as well as the software. Values for RBC and pRBC detected by ARAM are similar to the manually acquired results, shown in Fig. 4. Results for parasitaemia show significant agreement with the manual analysis, as is also confirmed by the Bland–Altman test (see Additional file 3: Figure S3). The error bars mark the statistical error which display the 90 % confidence intervals of the measures and overlap with the manually obtained results. ARAM detects a parasitaemia of 4.81 % whereas the operators count 4.75, 4.98 and 4.75 %. The statistical error is about 0.63 % each time. For the rosetting rate in Fig. 4 ARAM and the operator’s results are akin (ARAM 58.70 ± 6.70 %; operators 63.09, 59.03, 63.51 %). The rosette sizes are in good accordance within ARAM’s error range, although slightly larger deviations can be seen. The operators determine the mean rosette size as 3.87, 3.47 and 4.09 while ARAM gets 3.65 with an interquartile range of 1.39. The comparison of determined rosette size is not as obvious as the above mentioned measures due to technical reasons: ARAM only detects a two dimensional projection of the 3D object, like described above which leads to a less accurate calculation of rosette size than the calculation for the rosetting rate. Overall, the uncertainties are dominated by the statistical error in all cases here. For instance, to decrease the confidence interval to half of its width a sample size of about 12,500 RBC would be necessary, independent of the determination mode (manual or automated).

### Application of ARAM: rosette inhibition study

As an example for the application of ARAM, a study of the inhibitory effect of heparin on rosetting rate and size is performed. Three different heparin concentrations are chosen. Each sample is analysed by manual counting in the microscope, manual counting from the images (50 images each) and ARAM analysis (same 50 images each) to compare the results. Heparin interacts with the surface expressed *P. falciparum erythrocyte membrane protein 1* (PfEMP1) competing out the receptor on the RBC involved in the formation of rosettes. This leads to the disruption of already formed rosettes or inhibiting rosette formation [25, 26]. The detected parasitaemia is independent on heparin concentration within the confidence intervals, as can be seen in Fig. 5. The values are 6.21 ± 0.68, 7.84 ± 1.04 and 6.74 ± 0.88 %. The rosette rate is decreasing with increasing inhibitor concentration as analysed with any of the three methods, see Fig. 5 top right part. The differences between automatic and manual analysis of the images are much smaller than between manual image analysis and manual analysis directly performed with the microscope, probably due to the inhomogeneous distribution of RBC, pRBC and rosettes throughout the slide. By performing the analysis on a greater amount of samples this deviation is reduced, but without automation this is a time consuming task. The mean rosette size is greater for the specimen without inhibitor and so is the interquartile range of the rosette size distribution. The heparin treated specimens show smaller rosettes with a more narrow size distribution. The rosette size distribution (projection area) is depicted in the lower right part of Fig. 5. The trend of each measurement can be described by a rapidly decreasing function towards greater rosette size (positive x-axis). Additionally, a higher inhibitor concentration reduces the size of rosettes.

To the authors’ knowledge, currently there are no reports on rosette size distribution within in vivo or in vitro studies. Hence, the basic form of such a distribution is estimated with a simple model (see Additional file 4: Supplementary information). As argued, it is plausible to assume a Gaussian distribution of rosette size in terms of volume as indicated in Fig. 3 bottom bar chart. It is important to note that with the definition in Eq. 6 this Gaussian distribution is cut at the left side when there are non-zero values for rosette sizes smaller than the threshold. Since there are no studies about rosette size distribution, ARAM for the first time offers an easy way to analyse those and determine the amount of rosette objects necessary to get reliable and quantitative results of this highly important measure.

### Error analysis

**Fig. 6:**
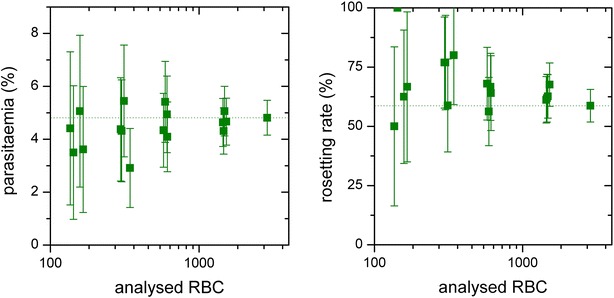
Sample size dependency. *Left* determined parasitaemia and *right* determined rosetting rate for randomly chosen sets of micrographs

Above the necessity of utilizing a statistical error is described. In Fig. 6 the impact of sample size on the results is exemplarily demonstrated. On the x-axis the count of analysed RBC is shown. The groups of measurement values correspond to the amount of RBC found on 5, 10, 20, 50 and 100 images. Obviously the width of the confidence interval and hence the statistical uncertainty of the experiment is reduced with increasing sample size for parasitaemia as well as rosetting rate. Especially for rare effects this dependency will be much more pronounced. With ARAM it is possible to gather such very rare effects, as it can realize a statistically significant amount of measurements with the necessary uniformity. Otherwise these effects can be simply rare or hidden in the measurement uncertainties. In order to get reliable conclusions, the amount of analysed RBC or images has to be large enough that rare effects are analysed in sufficient extend. ARAM offers the option to calculate the amount of images for desired confidence probabilities and width of confidence intervals from measured rosetting rate, number of analysed rosette objects and images. Assuming e.g. a value of interest v = 0.01 one would need 270 measurements to get ε = 0.01. And to get a reliable result with ε = 0.0023 a minimum of 5000 images has to be analysed.

### Image requirements

One major convenience of ARAM is the simplicity of performing an analysis: in a standard analysis the algorithm does not need additional input. Nevertheless, to ensure this simple operation the images have to meet some specifications. There obviously is a limitation for high cell density and low contrast. For the edge detection via Prewitt-filtering there needs to be a sufficient difference between the grey value of background and cell wall pixels and edges should not be blurred. For the detection of the pRBC, the fluorescence needs to be bright enough and clearly visible compared to the image background. To identify a cell cluster from the first analysis step (object detection with Prewitt-filter) as a rosette there has to be a detected pRBC and at least two RBC within the cluster. The size cut-off value m (see Eq. 6) can be adjusted in the configuration file. This might be necessary when rosettes appear too small and are therefore not detected.

The possibility to save the analysed images with rosettes and detected RBC marked, allows easy verification of the results by the operator and readjustment of the analysis with help of the configuration-file. The text export transferring and saving of the calculated values makes data collection easy and time-saving. Additional scripts could read those text files and create automated plots if desired.

**Fig. 7:**
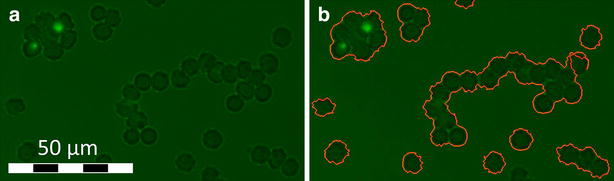
Cell density error. **a** Original image: the cell density is so high, that the non parasitized RBC are in contact and cannot be distinguished by the algorithm. **b** Analysed image: the cell agglomerate is detected as one object

The most important preparation parameter is the cell density on the micrographs. Hence, one has to account for a suitable haematocrit: if cells are too close to a cluster but not part of it they may be mistakenly detected as part of the rosette. To reduce this error the cell dilution of the specimen has to be chosen accordingly as illustrated in Fig. 7. Here the cell density is so high that non-bound RBC are in contact, leading to problems in single cell identification. This implies the intrinsic limitation of the presented algorithm.

Above-mentioned published software [17, 19, 21, 22] calculate parasitaemia from images, while [19] claim to additionally determine the parasites stage. ARAM not only calculates parasitaemia but also gives the statistical error to estimate the quality of the results. Beyond that ARAM is the only available software solution to analyse rosetting rate and rosette size distributions. The easy usage of ARAM is supported by its independence from other software, its graphical user interface and its results-text-export functionality, features the other solutions do not include. With these abilities ARAM is the only software suitable for laboratory work-flows to automatically analyse and characterize parasitaemia and rosetting in malaria infected specimens.

## Conclusions

Summing up ARAM produces accurate and reproducible results for parasitaemia, rosetting rate and rosette size distribution. This analysis is fast and performed automatically without the operators in depth knowledge of the software. Overall ARAM is a quick and simple way to reduce manual cell counting and produce reliable results for cell population monitoring and the study of drug influence on *P. falciparum* and its rosetting behaviour.

To improve the quality of measuring results and reduce time spent by the operator, the experiment can be further optimized, standardized and made operator independent. For now, the most time consuming part of this analysis is image acquisition. Consequently, a promising approach is the use of a motorized sample stage allowing to automatically vary the sample position within the slide, focus and take the images. Furthermore, real time analysis could use the existing functionality to determine the necessary sample size.

Concluding, ARAM has the evident potential to serve as tool in daily laboratory work as well as a platform for studies of so far inaccessible measures like precise rosette size distribution. All interested operators can download this new powerful tool for free from the following website: http://nanoquakes.de/ARAM/. ARAM is tested for 64-bit Windows © 7, 8.1 and 10.

## Additional files

10.1186/s12936-016-1243-4 Background-based algorithm. **a** shows the original image. **b** Displays the inverted grey value version of the original image. **c** The grey value matrix with subtracted background is shown. **d** Then shows the bottom left image with a threshold filter applied.
10.1186/s12936-016-1243-4 Threshold-based algorithm. **a** The original image. **b** The binary after threshold operation. **c** Original image with marked boundaries of detected objects. **d** Detected areas filled in.
10.1186/s12936-016-1243-4 Bland-Altman diagrams. Left Comparison of the cell detection by ARAM and an operator. Right Comparison of the determined rosette size by ARAM and an operator.
10.1186/s12936-016-1243-4 Supplementary information.

## References

[1] WHO. World Malaria Report. Geneva: World Health Organization; 2014.

[2] Miller L, Baruch D, Marsh K, Doumbo O (2002). The pathogenic basis of malaria. Nature.

[3] Adams Y, Rowe JA (2013). The effect of anti-rosetting agents against malaria parasites under physiological flow conditions. PLoS One.

[4] Silamut K, Phu NH, Whitty C, Turner GD, Louwrier K, Mai NT (1999). A quantitative analysis of the microvascular sequestration of malaria parasites in the human brain. Am J Pathol.

[5] Kaul D, Roth E, Nagel R (1991). Rosetting of *Plasmodium falciparum*-infected red blood cells with uninfected red blood cells enhances microvascular obstruction under flow conditions. Blood.

[6] Doumbo OK, Thera MA, Koné AK, Raza A, Tempest LJ, Lyke KE (2009). High levels of *Plasmodium falciparum* rosetting in all clinical forms of severe malaria in African children. Am J Trop Med Hyg.

[7] Carlson J, Helmby H, Wahlgren M, Hill A, Brewster D, Greenwood B (1990). Human cerebral malaria: association with erythrocyte rosetting and lack of anti-rosetting antibodies. Lancet.

[8] Newbold C, Pinches R, Roberts D, Marsh K (1992). *Plasmodium falciparum*: the human agglutinating antibody response to the infected red cell surface is predominantly variant specific. Exp Parasitol.

[9] Miller LH, Good MF, Milon G (1994). Malaria pathogenesis. Science.

[10] Rowe A, Obeiro J, Newbold CI (1995). *Plasmodium falciparum* rosetting is associated with malaria severity in Kenya. Infect Immun.

[11] van Hensbroek MB, Palmer A, Onyiorah E, Schneider G, Jaffar S, Dolan G (1996). The effect of a monoclonal antibody to tumor necrosis factor on survival from childhood cerebral malaria. J Infect Dis..

[12] Bull PC, Lowe BS, Kortok M, Molyneux CS, Newbold CI, Marsh K (1998). Parasite antigens on the infected red cell surface are targets for naturally acquired immunity to malaria. Nat Med.

[13] Ofori MF, Dodoo D, Staalsoe T, Kurtzhals JA, Koram K, Theander TG (2002). Malaria-induced acquisition of antibodies to *Plasmodium falciparum* variant surface antigens. Infect Immun.

[14] Staalsoe T, Shulman CE, Bulmer JN, Kawuondo K, Marsh K, Hviid L (2004). Variant surface antigen-specific IgG and protection against clinical consequences of pregnancy-associated *Plasmodium falciparum* malaria. Lancet.

[15] Cramer JP, Nussler AK, Ehrhardt S, Burkhardt J, Otchwemah RN, Zanger P (2005). Age-dependent effect of plasma nitric oxide on parasite density in Ghanaian children with severe malaria. Trop Med Int Health..

[16] Magistrado PA, Lusingu J, Vestergaard LS, Lemnge M, Lavstsen T, Turner L (2007). Immunoglobulin G antibody reactivity to a group A *Plasmodium falciparum* erythrocyte membrane protein 1 and protection from *P. falciparum* malaria. Infect Immun.

[17] Sio SWS, Sun W, Kumar S, Bin WZ, Tan SS, Ong SH (2007). MalariaCount: an image analysis-based program for the accurate determination of parasitemia. J Microbiol Meth..

[18] The MathWorks. MATLAB and statistics toolbox Release 2012; 2015.

[19] Diaz G, Gonzalez FA, Romero E (2009). A semi-automatic method for quantification and classification of erythrocytes infected with malaria parasites in microscopic images. J Biomed Inform.

[20] Rasband W, Image J (1997). US National Institutes of Health.

[21] Frean JA (2009). Reliable enumeration of malaria parasites in thick blood films using digital image analysis. Malar J..

[22] Tek FB, Dempster AG, Kale I (2009). Computer vision for microscopy diagnosis of malaria. Malar J..

[23] Moll K, Kaneko A, Scherf A, Wahlgren M. Methods in Malaria Research. http://onlineip.html5com/dcfj/sotp/ (2014). Accessed 22 Dec 2015.

[24] Henze N (2006). Stochastik für Einsteiger: eine Einführung in die faszinierende Welt des Zufalls.

[25] Vogt A, Winter G, Wahlgren M, Spillmann D (2004). Heparan sulphate identified on human erythrocytes: a *Plasmodium falciparum* receptor. Biochem J.

[26] Leitgeb AM, Blomqvist K, Cho-Ngwa F, Samje M, Nde P, Titanji V (2011). Low anticoagulant heparin disrupts *Plasmodium falciparum* rosettes in fresh clinical isolates. Am J Trop Med Hyg.

[27] Bland JM, Altman D (1986). Statistical methods for assessing agreement between two methods of clinical measurement. Lancet.

