# Supplementary figures and images for: ARAM: an automated image analysis software to determine rosetting parameters and parasitaemia in Plasmodium samples
Source: Malar J. 2016 Apr 18;15:223. doi: 10.1186/s12936-016-1243-4 (PMC4835829; doi:10.1186/s12936-016-1243-4)

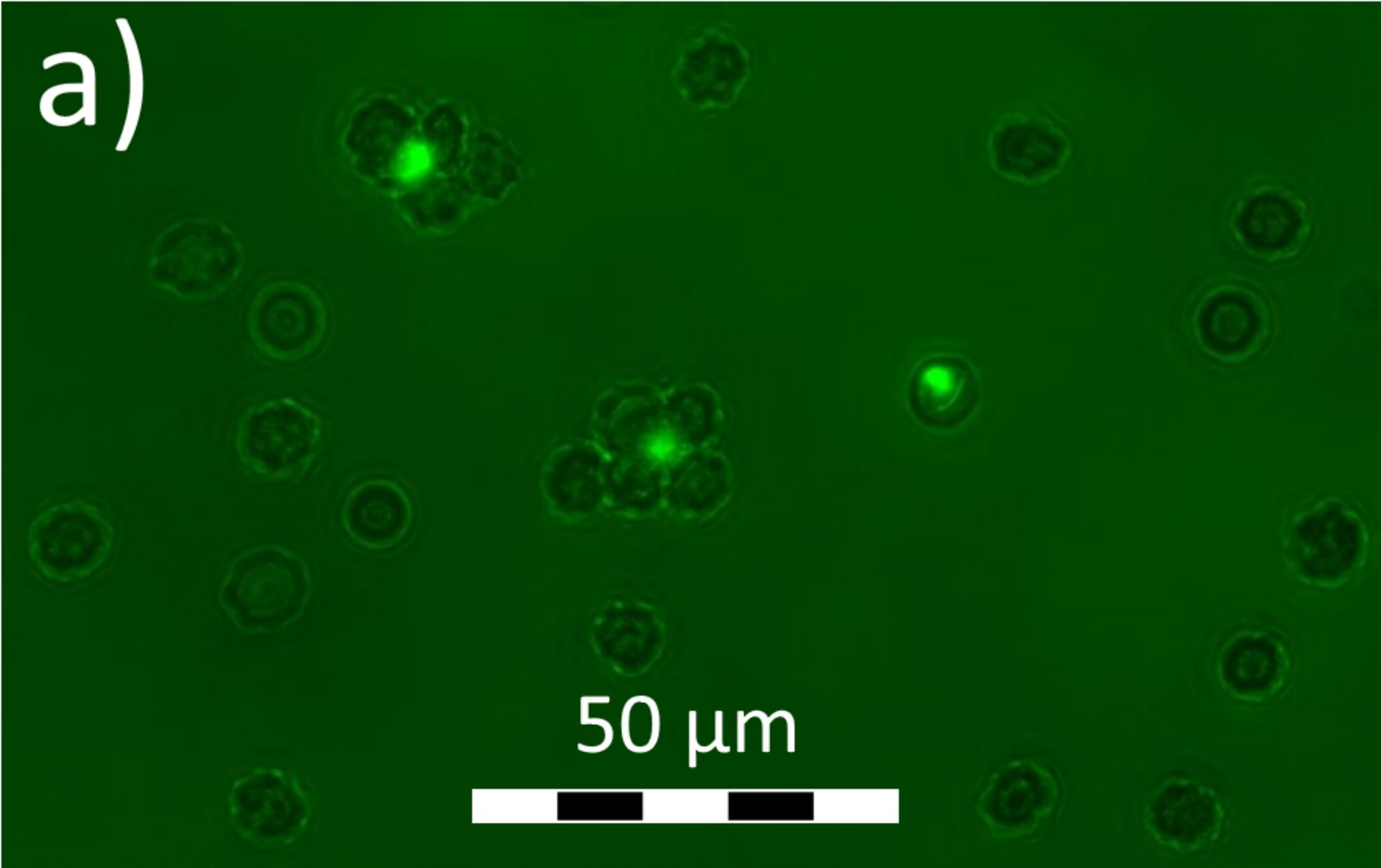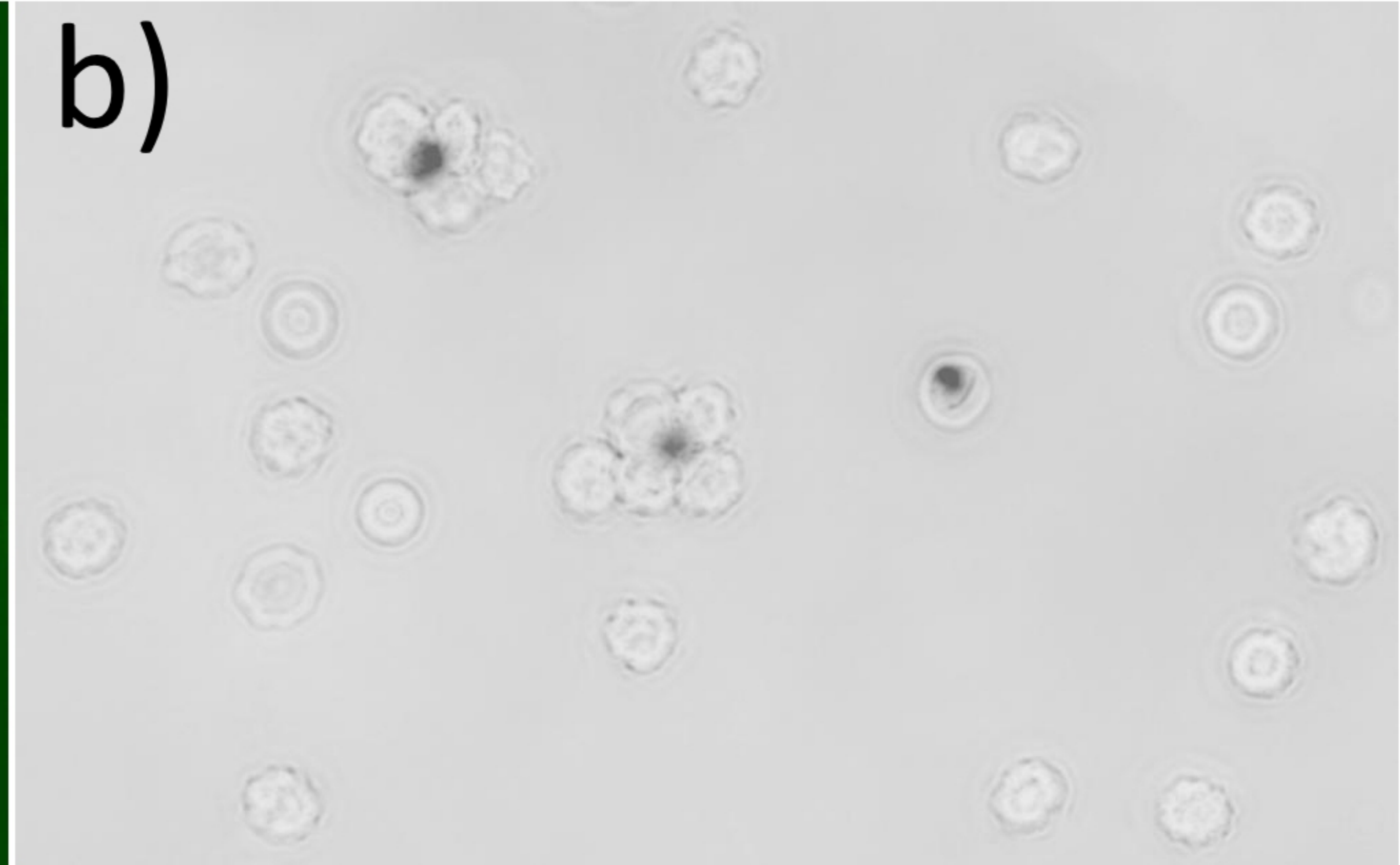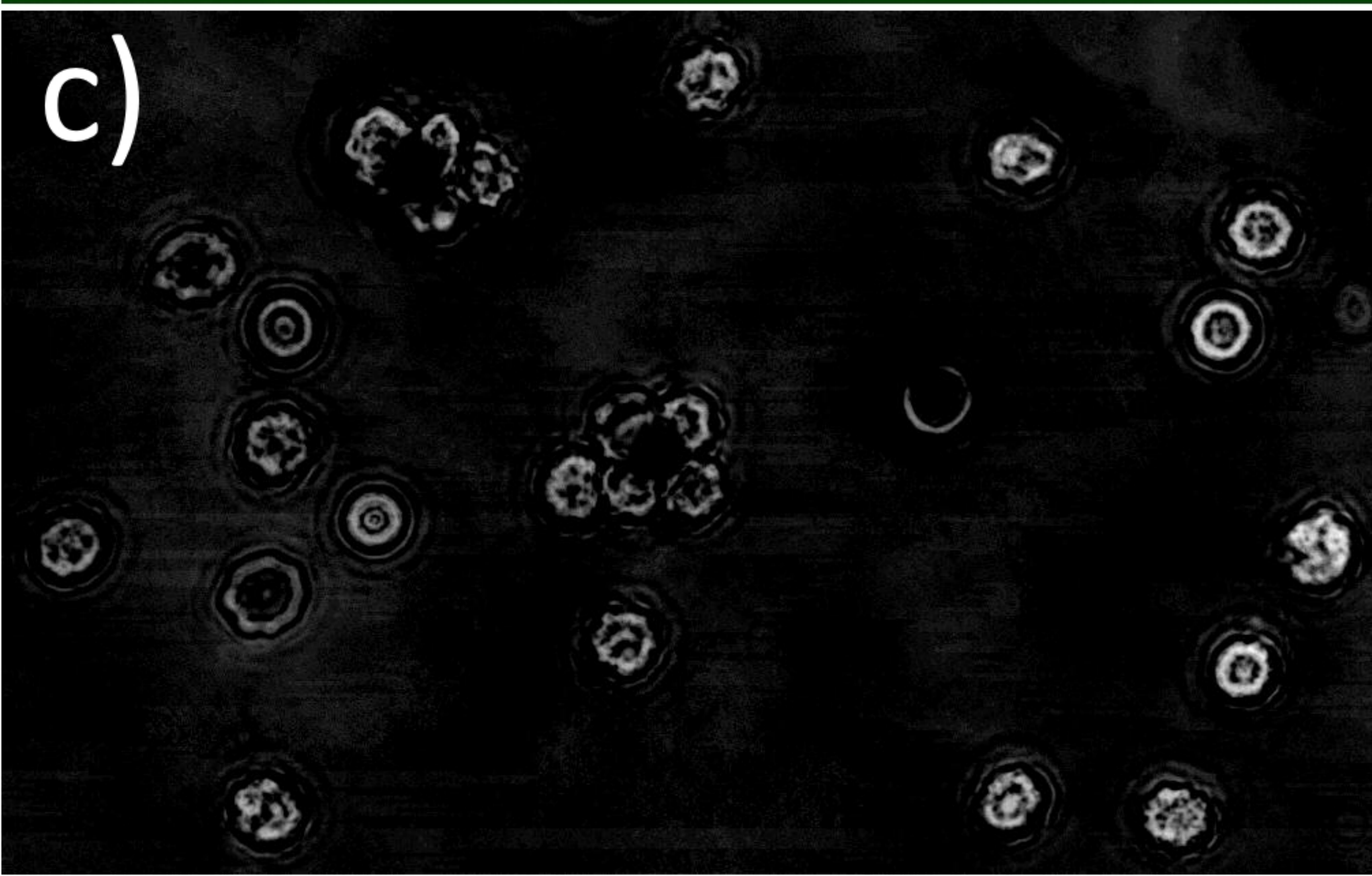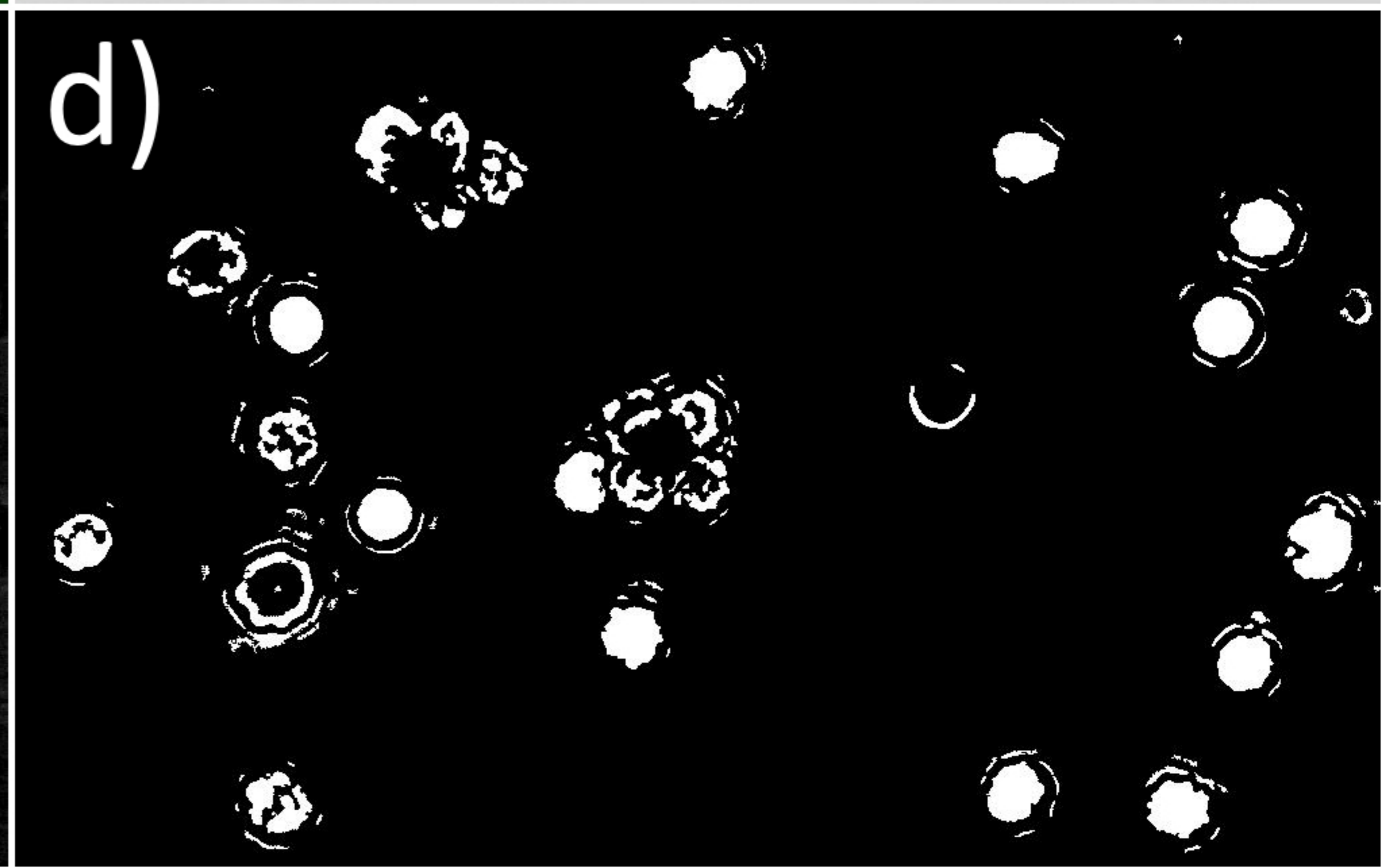

Supplement: Supplementary file 1 — 10.1186/s12936-016-1243-4 Background-based algorithm. a shows the original image. b Displays the inverted grey value version of the original image. c The grey value matrix with subtracted background is shown. d Then shows the bottom left image with a threshold filter applied. [file 12936_2016_1243_MOESM1_ESM.pdf]

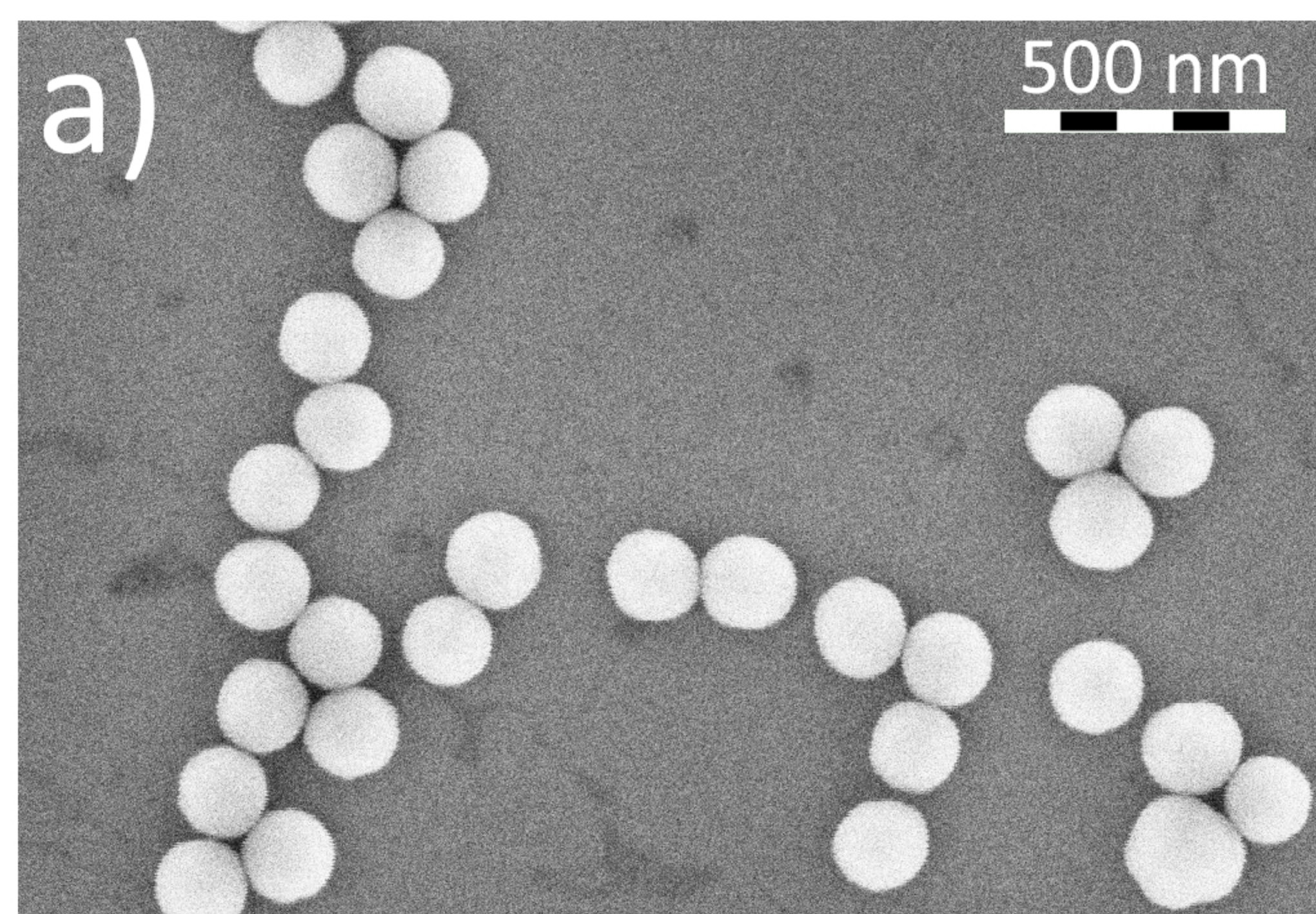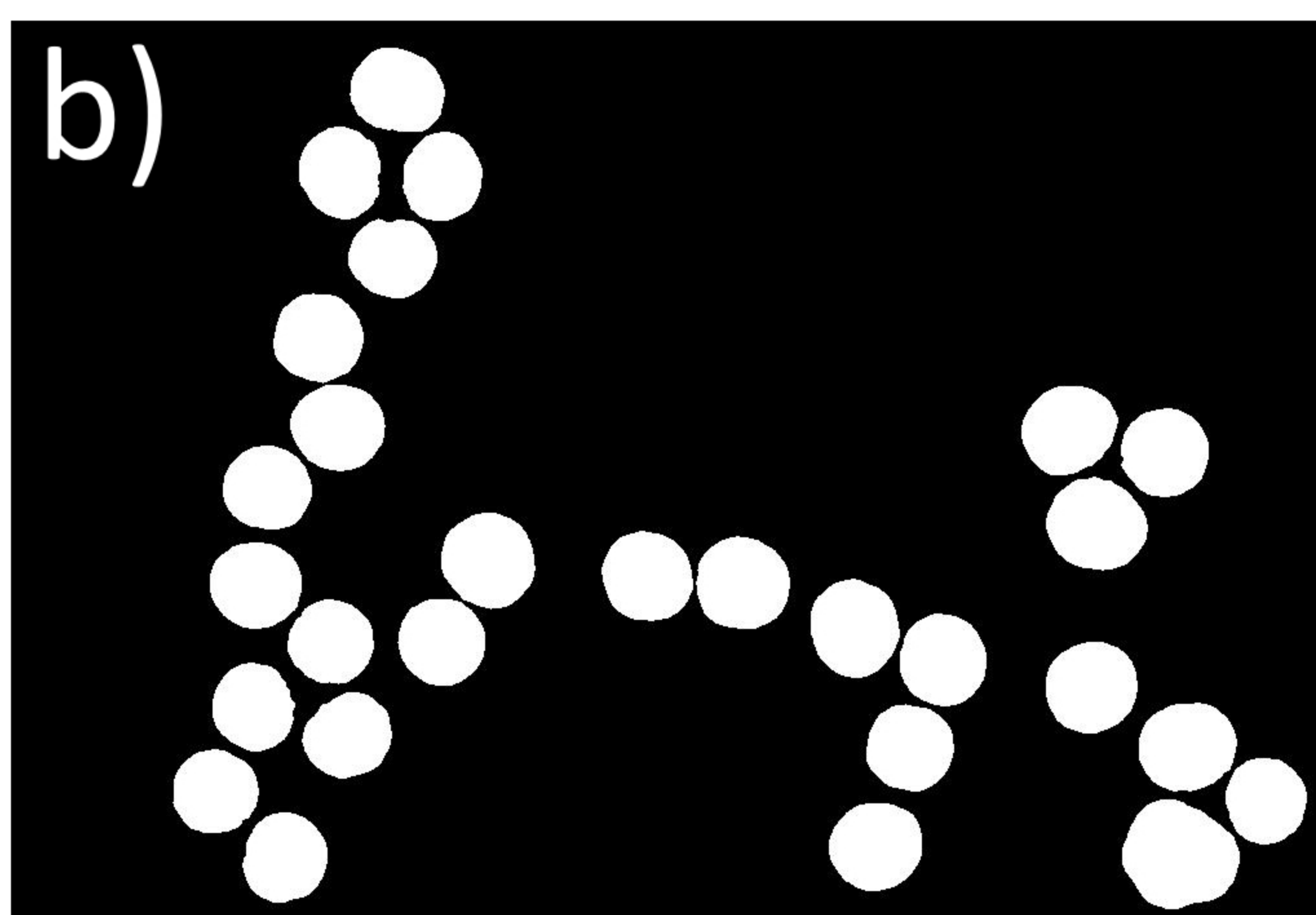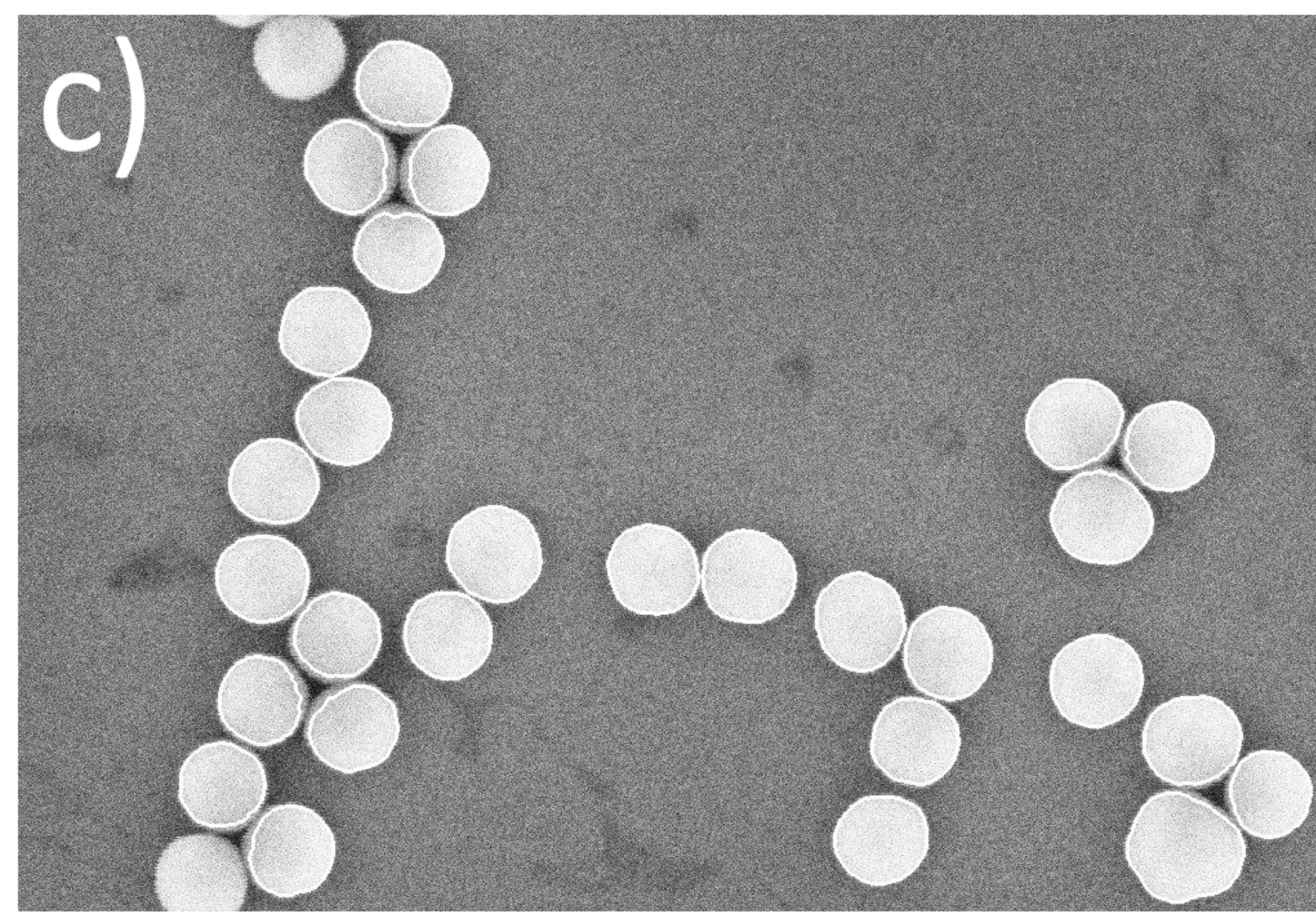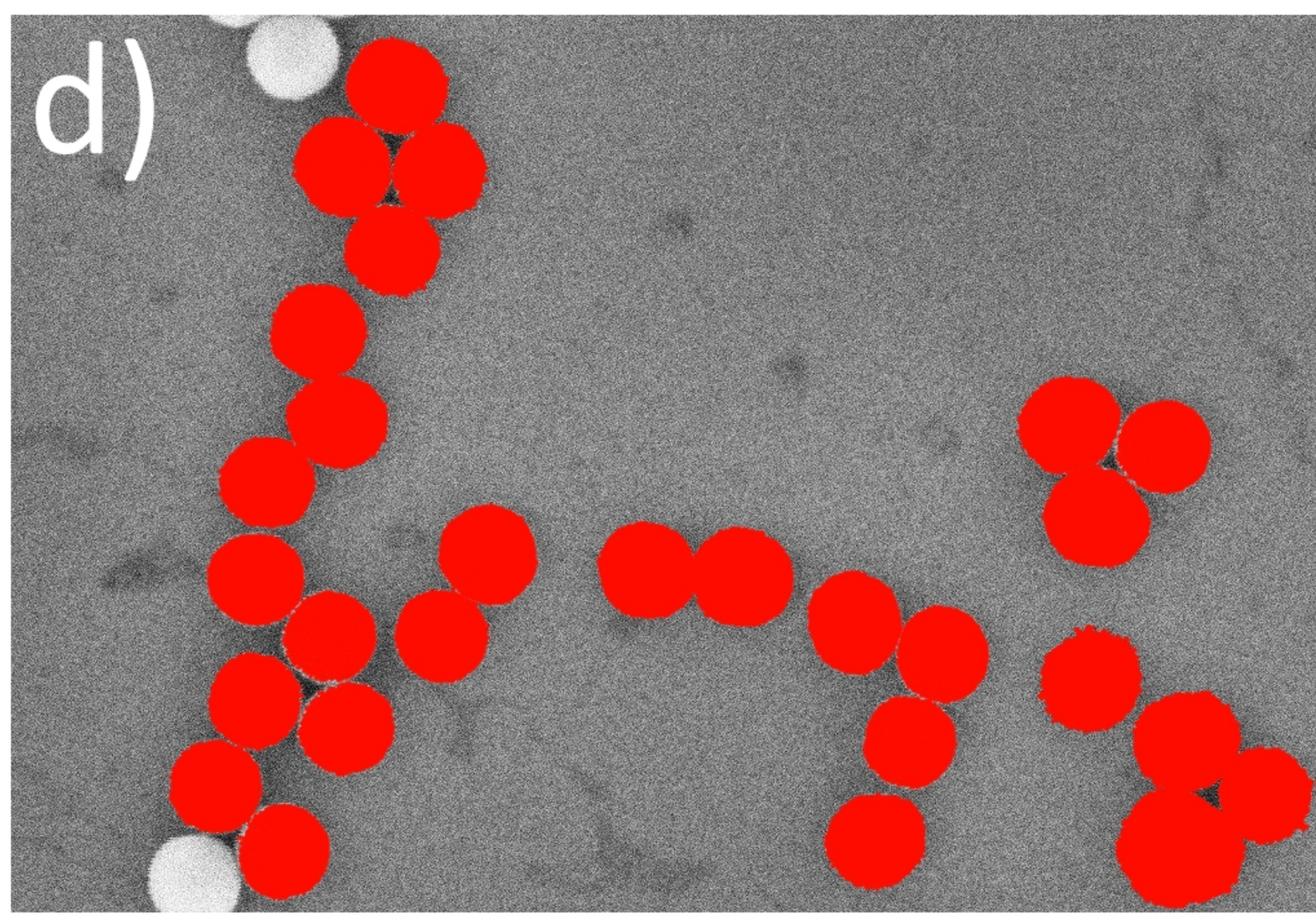

Supplement: Supplementary file 2 — 10.1186/s12936-016-1243-4 Threshold-based algorithm. a The original image. b The binary after threshold operation. c Original image with marked boundaries of detected objects. d Detected areas filled in. [file 12936_2016_1243_MOESM2_ESM.pdf]

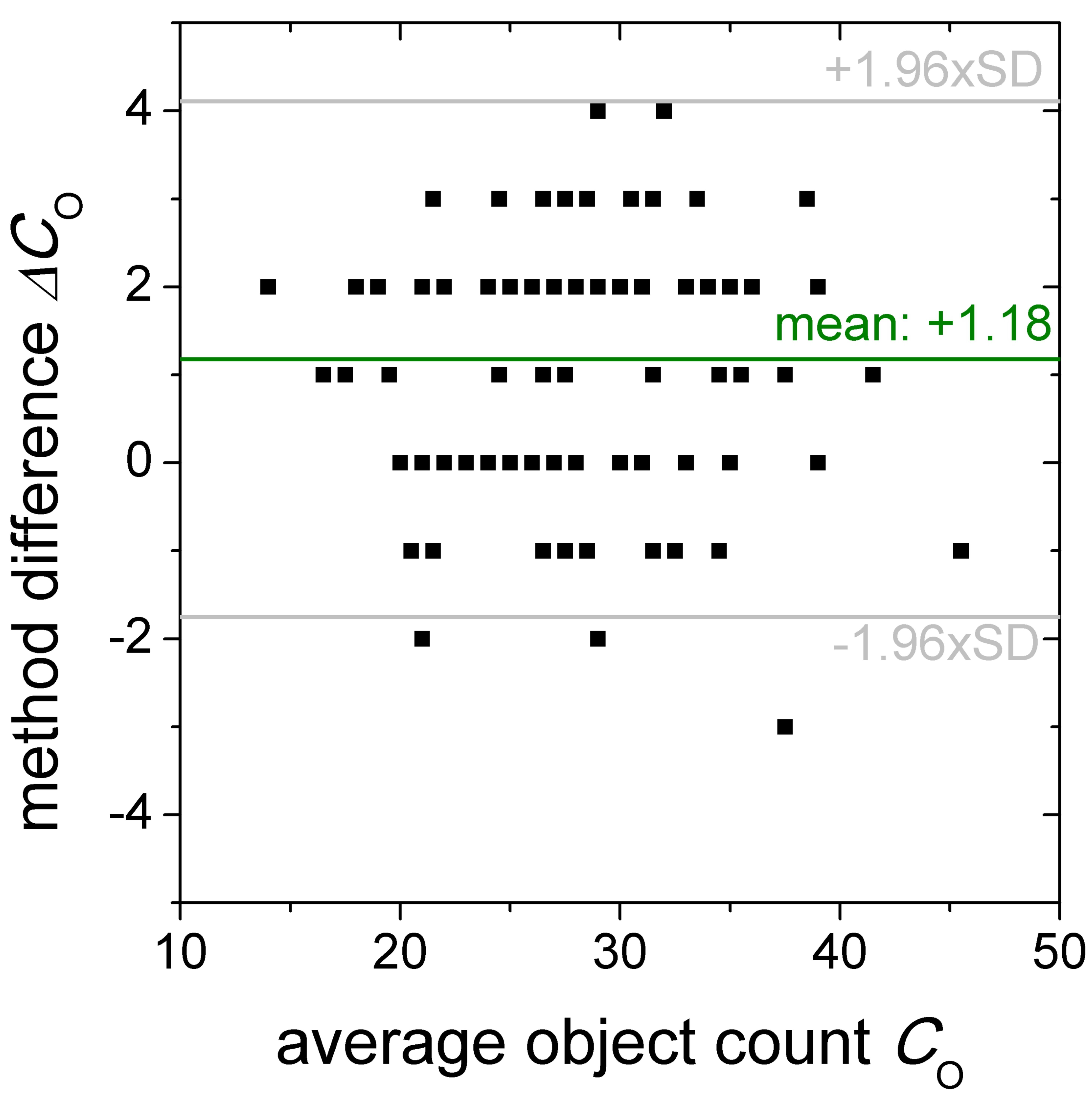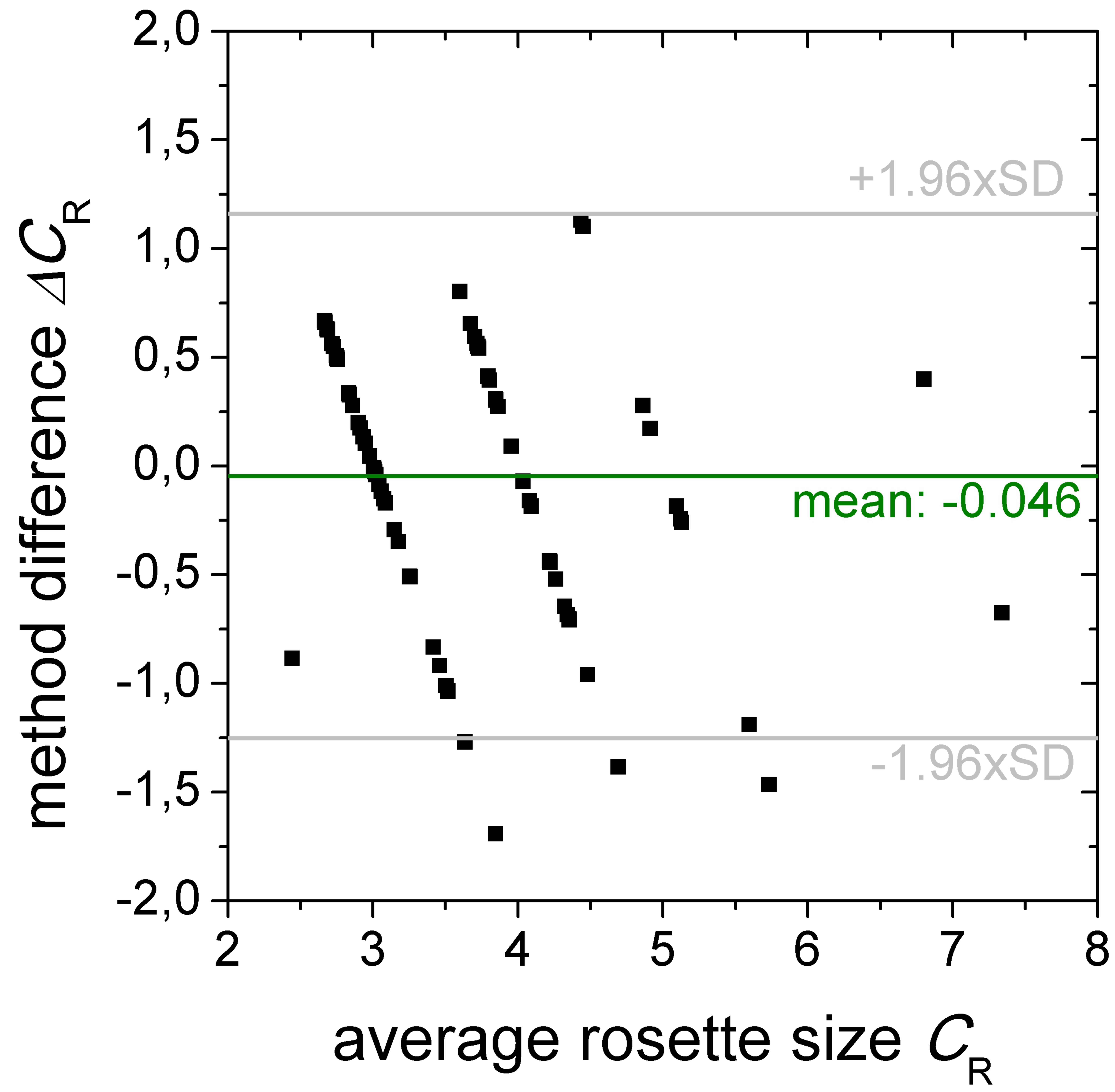

Supplement: Supplementary file 3 — 10.1186/s12936-016-1243-4 Bland-Altman diagrams. Left Comparison of the cell detection by ARAM and an operator. Right Comparison of the determined rosette size by ARAM and an operator. [file 12936_2016_1243_MOESM3_ESM.pdf]
