# Supplementary material for: ARAM: an automated image analysis software to determine rosetting parameters and parasitaemia in Plasmodium samples
Source: Malar J. 2016 Apr 18;15:223. doi: 10.1186/s12936-016-1243-4 (PMC4835829; doi:10.1186/s12936-016-1243-4)
Supplement: Supplementary file 4 — 10.1186/s12936-016-1243-4 Supplementary information. [file 12936_2016_1243_MOESM4_ESM.docx]

## Supplementary information to:

## **ARAM - an automated image analysis software to determine rosetting parameters and parasitaemia in Plasmodium samples**

### Rosette size distribution model

The basic form of a rosette size distribution is estimated with a simple model: In a test volume V of blood or cell

medium there are n RBC with the volume

$V_{\mathrm{RBC}_{\mathrm{total}}}=n \times V_{\mathrm{RBC}_{\mathrm{single}}}$

The haematocrit *h* is given by

$h= V_{\mathrm{RBC}_{\mathrm{total}}}/(V-V_{\mathrm{RBC}_{\mathrm{total}}})$

In first-order approximation a random distribution of RBC and diffusion of RBC

as the driving force of cell movement in the medium are assumed. For rosette formation a pRBC must be located in the vicinity of other pRBC or RBC. With typical diffusion constants for RBC at 37°C one now can estimate the relevant vicinity of a pRBC to collect RBC during rosette formation as a sphere with radius R. As diffusion constant D for a RBC the constant for an equivalently sized sphere (radius R_0_ = 4 µm, RBC disk diameter: 8 µm)

is assumed:

$D= \frac{k_{B}r}{6\pi\rho R_{0}}\approx\frac{1}{70}\frac{\mu m^{2}}{s}$ (10)

With a formation time *t* of 10 seconds the resulting typical mean path of diffusion *R* is

$R= \sqrt{6Dt} \approx10 \mu m$

The amount of relevant neighboring cells can be calculated by a simple binomial distribution. With the given haematocrit a Gaussian distribution with the expectation value γ is obtained. This is not the real rosette size but the potential rosette size in dependence of temperature and haematocrit. In a typical experiment ARAM and the operator can only analyse a 2D projection of 3D objects. Hence the rosette size distribution calculated by ARAM should have the shape of a Gaussian distribution with scaled x-axis: to get from rosette volume (e.g. a sphere) $V= \frac{4}{3}\pi r^{3}$ to a 2D rosette size $A= \pi r^{2}$ scaling with a factor $f\propto r^{\frac{2}{3}}$ is needed. This functionality is included in ARAM and can be accessed in the results window as indicated in figure 3.

### Background-based algorithm

In additional file 1 the background-based algorithm is shown. The most prominent structures are detected but intensity variations on a small scale cannot be filtered well. The resulting binary image has very disrupted structures and some are only partially detected.

### Threshold-based algorithm

As described above for some images a threshold based object detection delivers better results. In additional file 2 a micrograph of nanoparticles is analysed.

### Bland-Altman comparison of ARAM and operator counting

The Bland-Altman test visually compares the results determined by two different methods [27]. Here the results determined by ARAM and an operator are compared. Therefore, the graph *S*

$S\left( x,y \right)=S\left( C_{i},\Delta C_{i} \right)=S\left( \frac{V_{\text{operator}}+V_{\text{ARAM}}}{2}, V_{\text{operator}}-V_{\text{ARAM}} \right)$ (12)

is created. In additional file 3 the measures number of detected cell-objects and rosette size are compared. The structure in the right part of additional file 3 is due to systematic differences in the size determination of rosettes. While the operator determines the absolute number of cells in a rosette, ARAM detects the aggregate area which is a continuously distributed value. The rosette size’s method difference ∆*C*_R_ shows a good mean value of about zero with a symmetrical distribution of the measurements of ARAM and the operator. However, ARAM detects about 1 cell per image less than the operator (difference in the measurements shown in figure 4 ∼3.5%). This difference is based on the algorithm’s consequent exclusion of cells in contact with the micrograph borders, while the operators partly (and inconsequently) count such cells. The impact of this counting difference has only little impact on the final results.
